# Review of ten-years presence of *Aedes albopictu*s in Spain 2004–2014: known distribution and public health concerns

**DOI:** 10.1186/s13071-015-1262-y

**Published:** 2015-12-23

**Authors:** Francisco Collantes, Sarah Delacour, Pedro María Alarcón-Elbal, Ignacio Ruiz-Arrondo, Juan Antonio Delgado, Antonio Torrell-Sorio, Mikel Bengoa, Roger Eritja, Miguel Ángel Miranda, Ricardo Molina, Javier Lucientes

**Affiliations:** Departamento de Zoología y Antropología Física, Facultad de Biología, Universidad de Murcia, Murcia, Spain; Departamento de Patología Animal, Facultad de Veterinaria, Universidad de Zaragoza, Zaragoza, Spain; Department d’Agricultura, Servei de Gestió Forestal, Direcció General del Medi Natural i Biodiversitat, Ramaderia, Pesca, Alimentació i Medi Natural, Barcelona, Spain; Servei de Control de Mosquits, Consell Comarcal del Baix Llobregat, Parc Torreblanca, Sant Feliu de Llobregat, Spain; Departament de Biologia, Área de Zoología, Universitat de les Illes Balears, Palma de Mallorca, Spain; Unidad de Entomología Médica, Servicio de Parasitología, Centro Nacional de Microbiología, Instituto de Salud Carlos III, Madrid, Spain

**Keywords:** *Aedes albopictus*, Spain, Vector-borne diseases, Chikungunya, Dengue, Vector map, Review

## Abstract

Ten years have gone by since the Asian tiger mosquito *Aedes albopictus* was recorded for the first time in Spain. In this paper, all relevant published information about this vector in Spain for the period 2004–2014 is reviewed. The known distribution for 2014 is provided, including all historical records (published and unpublished data) and the results from samplings of the last year. The consequences on public health about the presence of the Asian tiger mosquito in Spain are also highlighted. Further, legal aspects and control plans related to the management and diseases transmitted by this invasive vector species are also discussed.

**Electronic supplementary material:**

The online version of this article (doi:10.1186/s13071-015-1262-y) contains supplementary material, which is available to authorized users.

## Background

*Aedes albopictus* (Skuse, 1894) (Diptera; Culicidae) is an invasive species whose first detection in Europe occurred in Albania in 1979 [1]. At present, this species has been recorded in 22 countries of Europe, even if it is considered established only in 15 as a result of different and continuous arrivals [2].

In 2004, this species was recorded in Spain for the first time [3] and ten years after that it was founded in almost all Mediterranean Spanish coast, as it is shown in this work.

The objective of this review is to compile all published information about *Aedes albopictus* for Spain and update the known distribution of this vector in Spain until 2014. In this way, we deal with all information about distribution but, in addition, we review other related aspects with perspectives of public health impact, legal situation and control measures.

## Review

### Arrival of *Aedes albopictus* to Spain

Although the source of arrival to Spain remains unknown, the historical entrance to the neighbouring country, France, is well documented. The first detection was in used tyres storage centres in the north of France in 1999 [4] and successive years 2002, 2004 and 2006, being originated from international trade of used tyres from USA [5]. However, nowadays the main established populations are in the Mediterranean France, where *Ae. albopictus* was recorded for the first time in 2004 [5]. At that time, the risk of introduction to Spain from France was considered to be high [6] and the EVITAR network (network for the study of viruses transmitted by arthropods and rodents financed by the Spanish Ministry of Health) included the surveillance of this species as a priority. In this sense, a thoroughly surveillance conducted in used-tyres storage centres from 2003 to 2004 did not show its presence [7]. Nevertheless in 2004, an increase on residents complains about insect bites was recorded in Sant Cugat del Vallès (Baix Llobregat, Catalonia). An entomological study conducted in the area showed the presence of *Simulium ornatum* (Meigen, 1818) and, for the first time in Spain, also the presence of *Ae. albopictus* [3, 8]. It is highly probable that *Ae. albopictus* was in the area at least during the two previous years, based on the records about biting incidence on the residents [8]. As we have noticed in our regular fieldwork, the resident's perception about the presence of the tiger mosquito begins when it is already established for one to two years.

### Surveillance and known distribution in Spain till 2014

#### Surveillance in Spain: history and situation

It is worth mentioning that the majority of the Spanish territory has not been systematically surveyed yet and the positive areas have not been continuously monitored. There was not and there is not, at present, any initiative at country level to coordinate the different programs and projects about surveillance of tiger mosquito. After the first detection in 2004, several occasional studies, some of them extensive but never systematic, have been carried out to find *Ae. albopictus* in Spain.

Because Catalonia was the first colonized and most affected region, the sampling has been more intense and continuous and several local institutions have contributed to the surveillance, namely: *Generalitat de Catalunya, Servei de Control de Mosquits del Consell Comarcal del Baix Llobregat, Agència de Salut Pública de Barcelona* and *Servei de Control de Mosquits de la Badia de Roses i Baix Ter* [9–13]. The Valencian Community (located south of Catalonia) has been also repeatedly sampled by different research groups. The research team of the Univerisity of Valencia have provided some isolated records and opinion articles about *Ae. albopictus* [14–23].

In addition, the Spanish Ministry of Health, Social Services and Equality [Ministerio de Sanidad, Servicios Sociales e Igualdad] has sponsored since 2007, the contract *“Entomological surveillance at airports and ports against imported vectors of exotic infectious diseases and surveillance of potential indigenous vectors of such diseases”*, which has enhanced the surveillance of *Ae. albopictus* in important ports and airports of mainland Spain, as well as in the Balearics and Canary Islands, and the Spanish Mediterranean side, exclusive Catalonia [24–34].

The majority of the above mentioned active surveillance has been conducted by means of oviposition traps (ovitraps) and to a lesser extent, by using BG-Sentinel™ traps (Biogents AG, Regensburg, Germany), CDC-type light traps, larval sampling and human landing caches. However, no comparison of the efficacy of the different methods has been conducted in Spain. The use of standardise methods, either for detection and monitoring of invasive mosquitoes species, at national and international level (i.e.: EU), appears as an appealing challenge for researchers and public health officers. As an example, current research groups in Spain use different types of ovitraps (different in volume and size of opening), as well as different density of traps and sampling frequency. Except for the first detection of the Asian tiger mosquito in Balearic Islands [32] and some parts of Catalonia, the European Centre for Disease Prevention and Control (ECDC) guidelines [35] and WHO-EMCA guidelines [36] have not been followed for the rest of Spain.

In addition to active surveillance, an innovative passive mosquito surveillance based on citizen’s observations has been developed in Spain in similar way to other countries in Europe [37]. The project AtrapaelTigre.com (“*hunting the tiger*”) allows non-expert residents to upload, by means of a phone application, pictures of mosquitoes linked to a geo-referenced locality. Afterwards, those pictures are validated by experts that indicate the potential presence of *Ae. albopictus* in the locality, which will be confirmed by means of field sampling. Thanks to this project an unexpected isolated population in the Malaga province was detected and the Andalusia region was included in the list of colonised areas [27]. The ECDC [38] recommends to include this type of initiatives in the plans of mosquitoes control.

#### Known distribution till 2014

In this section, we have compiled information about all Spanish municipalities where *Ae. albopictus* has been recorded from 2004 to 2014. The data have been originated from published studies, unpublished data of surveillance made from the Catalonian administration and the results from 2014 of the above mentioned *“Entomological surveillance at airports and ports against imported vectors of exotic infectious diseases and surveillance of potential indigenous vectors of such diseases”*. The field studies were done under the with institutional, regional and national guidelines/laws and special approbations for ethics committees were not necessary.

**Fig. 1:**
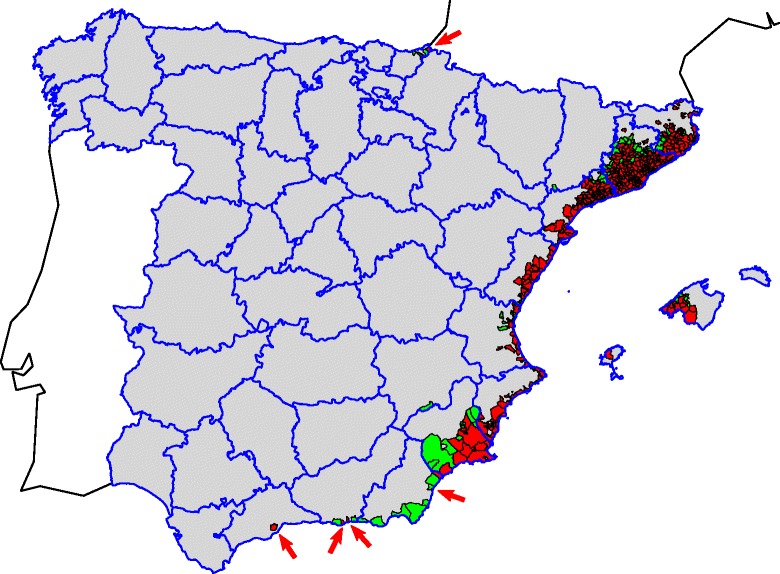
Known distribution of *Aedes albopictus* in Spain in 2014. *Red*: recorded in municipality; *Green*: not recorded; *Gray*: not studied. The *arrows* mark small positives isolates municipalities

From 2004 to 2014, *Ae. albopictus* has been recorded in 470 municipalities belonging to 13 provinces from six autonomous communities in Spain (Table 1, Fig. 1), highlighting the recent addition, in 2014, of 2 regions and four provinces (Andalusia: Málaga, Granada and Almería; Basque Country: Guipuzcoa). The record in Maials (Lleida province, Catalonia), in 2008 [10], is considered as an sporadic record, since only adults were recorded once (in a tyre dump which was subsequently disinsected), and not established populations were detected in subsequent years. The very recent finding in the Basque Country [15], considered as an early detection, has lead to set up an intensive surveillance in this area to check for population establishment and the undesirable starting of the colonisation of the Spanish Atlantic side.

**Table 1:** Resume of positive municipalities recorded for *Aedes albopictus* in Spain. The record in Lleida (Maials, 2008) is considered as an sporadic record

	Catalonia					Valencian Community				Balearic Islands		Region of Murcia	Andalusia			Basque Country	
Record year	Barcelona	Girona	Lleida	Tarragona	T	Castellón	Valencia	Alicante	T	Balearic/Majorca	Balearic/Ibiza	Murcia	Almería	Granada	Málaga	Guipúzcoa	T
2004	2				2				0								2
2005	8			1	9			1	1								10
2006	17				17				0								17
2007	27				27				0								27
2008	24	5	1	4	34				0								34
2009	28	7		2	37			1	1								38
2010	37	2		11	50	1			1								51
2011	23	28		19	70	1		2	3			1					74
2012	18	8		20	46	5		4	9	5		2					62
2013	10	1		11	22	15	4	11	30	2		3					57
2014	15	18		25	58	1	10	9	20	5	1	8	2	2	1	1	98
Total	209	69	1	93	372	23	14	28	65	12	1	14	2	2	1	1	470

**Fig. 2:**
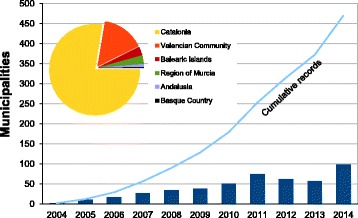
Evolution and amount of positive recorded municipalities for *Aedes albopictus* per year in Spain 2004–2014. Line: Cumulative records

**Fig. 3:**
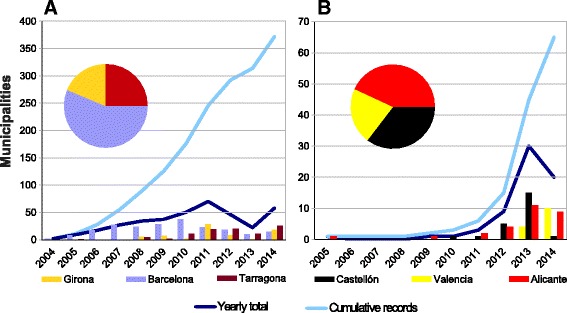
Evolution and total amount of positive recorded municipalities for *Aedes albopictus* per year in Spain. **a** Catalonia 2004–2014; the record in Lleida (Maials, 2008) is not included due to it is considered as an sporadic record. **b** Valencian Community 2005–2014

**Fig. 4:**
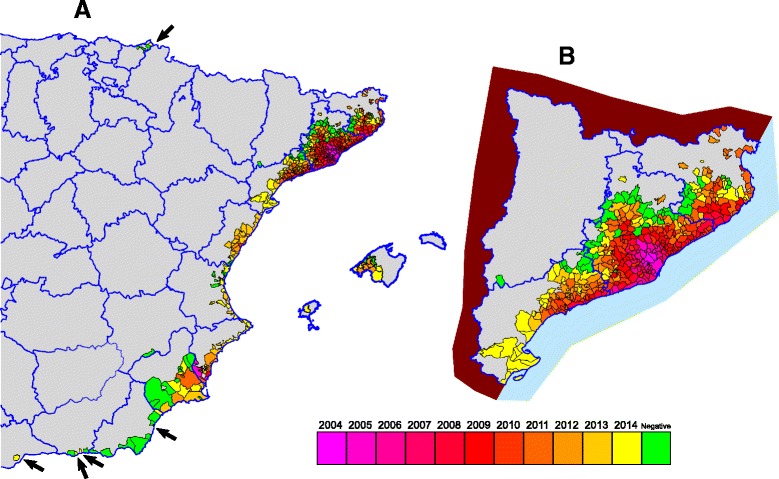
Historical record of positive municipalities by years (2004–2014). **a** General. **b** Detail in Catalonia. The *arrows* mark small isolated positives municipalities. *Green* color means negatives in 2014. *Gray* color means that these areas were never studied

During the last ten years, a remarkable rise of the *Ae. albopictus* distribution, mainly in Catalonia and Valencia Community, of mainland Spain has occurred (Figs. 2 and 3). It has not only increased, but an acceleration of the process has been observed. The historical records for every municipality, with their references as well as the new records are shown in the Additional file 1: Appendix and Fig. 4. Furthermore, *Ae. albopictus* was not detected in the provinces of Zaragoza (airports) and Huelva, where other mosquitoes are routinely sampled, but specific methods for tiger mosquito, such as ovitraps, are not used.

In regards to the Spanish archipelagos, *Ae. albopictus* has been recorded from Majorca [32] and Ibiza [34], whereas it is absent in the Canary Islands where an active surveillance is carry through in ports and airports of Gran Canaria, Tenerife and La Palma, since 2013 [39].

Because the majority of Spain has not been studied yet, as it is said above, we believe the known distribution, shown in the present work, could easily extend in the next years. During these ten years, several risk models based on climatic factors have propounded maps for Europe where areas of the Southern Spain would be less suitable to be colonised (even 0 % of suitability in some models) by *Ae. albopictus* [2]. However, over the last decade, *Ae. albopictus* was detected, firstly in some isolates areas until it has colonized almost all the Spanish Mediterranean Arch, from Northern Catalonia to Málaga. As Collantes et al. [40] pointed out in 2014, these risk models have included mainly climatic macro-variables, such as precipitation. Other variables, such as social ones, are generally not considered in the models, for example, water-saving culture due to usual drought in Southern Spain, makes available numerous artificial containers that are larval habitats for *Ae. albopictus* regardless precipitation. Roche et al. [41] included the land use (social variable) in the recent study of spread of this vector in France, which is an important factor of prediction of establishment.

The sequence of occupation of *Ae. albopictus*, since its detection in Catalonia in 2004, shows a pattern of colonisation from the coastal area to inland in mainland (Fig. 4), which could fit with a diffusion process. However, some jump dispersals have been observed in other areas. The first and best known happened in Orihuela municipality (Valencian Community), which is located almost 500 km from the contemporary *foci* of Catalonia [11]. The owner who found and identified the tiger mosquito in her vacation house was resident in Sant Cugat del Vallès, one of the first recorded localities in Spain. For this reason, it was speculated about the importance of the passive transport inside cars, and it was taken into account the surveillance around the highway A-7, the major road which runs along the Spanish Mediterranean coast, based on the hypothesis from Moore & Mitchell [42] in USA. Recently in France, Roche et al. [41] assumed this hypothesis too and they found dispersal jumps but considered that the most of them have not started new fronts, though the temporal sequence of positives departments in France shows gaps between them (http://ecdc.europa.eu/en/healthtopics/vectors/vector-maps/Pages/VBORNET_maps.aspx).

Some details about the environmental variability of known distribution of tiger mosquito in Spain can be pointed to. It has been found in a broad altitude range. There are many points almost at sea level and the highest records were in the Castellterçol (713 mSL) and Berga (702 mSL) municipalities (Barcelona, Catalonia).

At the moment, studies to infer correlation between environmental factors and the distribution of *Ae. albopictus* have not been published in Spain and the inadequate budgets have been channelled towards sampling from anthropized/urbanized areas. Now, some of us are currently involved in a project with this objective.

**Fig. 5:**
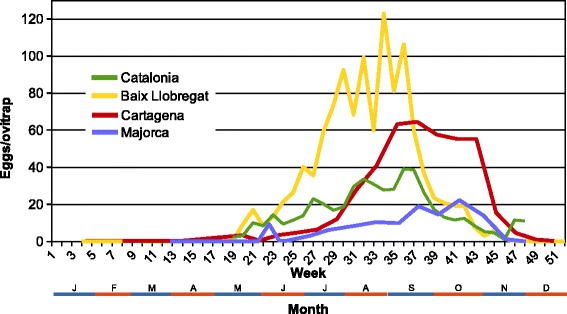
*Pseudo* phenology of *Aedes albopictus* as eggs/ovitrap along the time (weeks of year). Catalonia: Data from the Generalitat de Catalunya; mean number of eggs/ovitrap from positive municipalities in the same week of the year (2005–2014). Baix Llobregat: Data from the Consell del Baix Llobregat (2006–2014); mean number of eggs/ovitrap in the same week of the year. Cartagena: number of eggs/ovitrap from positive localities in 2014, in the municipality of Cartagena (Region of Murcia). Majorca: number of eggs/ovitrap from positive localities in 2014, in the island of Majorca (Balearic Islands)

In the same sense, due to meagre budgets, only in some areas, continuous surveillance has been carried out. The longest record is from Catalonia but some data of seasonality has been also recorded in parts of the Balearic Islands and the Region of Murcia. Because the ovitraps sampling has been the most used method in Spain, we have not got direct data of density. But, as some authors have already pointed out (Facchinelli et al. 2007, Abramides et al. 2011, etc.), it could be inferred from the oviposition catches or the number of positive points since there is correlation between the number of positives and the number of eggs in the same date: R^2^ = 0.94 (Cartagena); R^2^ = 0.92 (Majorca). These pseudo phenologies are shown in Fig. 5: General phenology in Catalonia (the number of municipalities has changed along this period) from data of the Generalitat de Catalunya, between 2005–2014; phenology of Baix Llobregat (Barcelona province) from data of Consell del Baix Llobregat. From this pair of datasets, the first captures in Catalonia are placed on the 17^th^ week of the year and the last one on the 78^th^ week. Only for 2014, we have got almost the full annual cycle from Balearic Islands (Majorca) and Region of Murcia (Cartagena municipality). In Majorca the first and last records were on 4/VI/2014/ and 3/XII/2014. In Cartagena and another point of the region (in Murcia municipality [40]) we found a few positives samples with few eggs or larvae even in wintertime.

When comparing the four curves, the population peaks shift to the end of year in the southern places, although the first appreciable increase (not the first record of the year) is near of the middle of May for all.

### Public health impact

*Aedes albopictus* is a daytime biting mosquito and shows an aggressive anthropophilic behaviour. In Barcelona, it was found 100 % of anthropophilic preference for *Ae. albopictus* after analysing the origin of ingested blood [43]. In addition, this species also bites other vertebrates, depending on hosts availability, thus enhancing the transmission of zoonoses when acting as a bridge between animals and humans [44–46]. Obviously, this marked feeding preference has important consequences for the public health. To date, 27 arboviruses belonging to 5 families [47, 48], as well non-identified flaviviruses [49], have been isolated from *Ae. albopictus.* Although its vector role is uncertain for the majority of them, the tiger mosquito has been clearly demonstrated as vector of dengue (DENV) and chikungunya (CHIKV) viruses [48]. In fact, *Ae. albopictus* was responsible of the recent European autochthonous cases of dengue in France (2010, 2013, 2014) and Croatia (2010) [1, 50], as well as chikungunya in Italy (2007) and France (2010, 2014) [1, 51]. Also, this species is vector of both species of nematodes *Dirofilaria repens* Railliet & Henry, 1911 and *D. immiti*s (Leidy, 1856) [44, 52].

The last cases of autochthonous dengue in Spain occurred in the first half of the twentieth century and they were associated to the presence of *Aedes aegypti* (L., 1762) [53]. In 2013, the Spanish Centre for Health Alerts and Emergencies [Centro de Coordinación de Alertas y Emergencias Sanitarias (CCAES)] issued a risk analysis about dengue [53] and concluded that the risk of local transmission in Spain would be low. However, imported cases of dengue were recorded in almost all autonomous communities of Spain. Geographically, the highest amount of cases was reported from Catalonia and Madrid, followed by Basque Country and Valencian Community. Moreover, the maximum number of cases was reported during August and September. From 2004 to 2014, the highest number of imported cases peaked in 2010, in coincidence with the observed maximum of cases in the countries of origin at that time [54]. Imported cases of dengue pose a risk of local transmission in Spain, as was already mentioned for France. Catalonia is the region were both the higher number of imported cases and the widest distribution of *Ae. albopictus* have been reported, thus showing the higher risk of local transmission of dengue. Besides that, the distribution of *Ae. albopictus* is currently expanding from year to year (Fig. 4), increasing even more the risk of local transmission.

In regards to chikungunya, imported cases from India into Spain are more recent (2006) and scarce [55, 56]. Beside that, the numerous cases of chikungunya reported from the Caribbean in 2013 [57] supposed an increase of imported cases into Spain [56, 58]. Also due to the health alert declared in France [59], a rapid risk assessment about chikungunya was conducted in Spain. The risk level was considered as medium due to the numerous cases in the Caribbean Islands, the high frequency of flights connections between both regions and the presence of *Ae. albopictus* in Spain, however, no autochthonous cases have been detected to date. The cumulative number of confirmed cases of imported chikungunya into Spain was 266 in 2014, most of them (96 %) from Latin America, mainly from the Dominican Republic (69 %). 70 of these confirmed cases were detected in Catalonia, where *Ae. albopictus* is well established. This is a growing concern from the point of view of public health [60].

Winter activity of *Ae. albopictus* adults is another important aspect when considering the risk of local transmission in Spain. In Southern Spain, Collantes et al. [40] recorded, for the first time in Spain the winter reproductive activity of adults. These findings possess important epidemiological consequences, mainly due to the increase of population dispersal and the active period for local transmission. Then, temperature could be less important as limiting factor for colonisation in this latitude than in northernmost areas, and others factors, as water availability, could become more relevant.

In Spain, the epidemiological surveillance of notifiable diseases is regulated by the Spanish law 2210/1995 [61]. Neither dengue nor chikungunya were included in the original list of 1996. In 2013, a new catalogue for notifiable diseases [62] was updated to be used by the Spanish Epidemiological Surveillance Network [Red Nacional de Vigilancia Epidemiológica (RENAVE)]. The protocols for chikungunya fever, dengue and West Nile virus diseases were included, however, the list of notifiable diseases was not legally updated until March 2015 [63]. In consequence, there was a period of two years (2013–14) when imported cases of dengue and chikungunya could be underestimated due to the lack of a legal framework for arthropod borne notifiable diseases.

Finally, no autochthonous cases of dengue or chikungunya have been detected in Spain. Apart of under-reporting due to sub-clinic courses, two hypotheses could be formulated but remain unsolved yet. The number of imported cases is low in Spain (1); the density of mosquitoes is still low (2). In Spain, there were less imported cases than France, where authochtonous cases of chikungunya have occurred. Although, the number of imported cases has been correlated with the number of autochthonous cases of dengue in Brazil and Australia [64, 65], the last autochthonous cases of chikungunya in France come from a primary case with African origin, even if the imported cases from the Caribbean represent 90 % of total detected in France [51].

About the relationship between density of vectors and health risk, all samplings conducted in Spain are mostly absence/presence ones, which means that there is no abundance maps available from the different Spanish regions where *Ae. albopictus* is established. As it is said above, the usual methodology of sampling has been by ovitraps in non standardised nor systematic way, so it is difficult to assign population densities in the studied areas. Therefore, it is difficult to link risk of transmission with mosquito density in areas where imported cases are frequently detected. However, for dengue, the relationship between vector density and transmission has not been sufficiently established or known neither and it is possible that transmission could occur under low densities of vector if the human population immunity (to this disease) is low [66].

At present, the health impact of *Ae. albopictus* in Spain is restricted to the biting nuisance. Several studies have shown that the mosquito nuisance affects to health (even without diseases transmission), quality of life and economy [67–69]. In Spain, few studies on this subject have been carried out. The biting incidence was studied after the first detection in Spain [70, 71], showing a raise of health and social problems as, for example, raise of bites and lesions, reduction of quality of life, disputes between neighbours and public unrest. Guasch [72] tried to quantify the economic impact of the biting nuisance in the municipality of Rubí (Barcelona, Catalonia) by means of the Choice models methodology (Choice experiment: Willingness to pay (WTP)). This study concluded that 60 % of interviewees would accept to pay the highest amount proposed, 10 € by year, to reduce the populations of *Ae. albopictus*.

### Legal situation of interventions on *Aedes albopictus*

The Asian tiger mosquito is an invasive exotic species and its presence in Spain is regulated by the Spanish law on invasive species [73]. This legal framework has two remarkable consequences. First, despite its health implications, the responsibility for its control lays on environmental authorities instead of health authorities. Second, the law forbids or limits its capture, possession, transport and commerce. Then, in a strictly legal framework, a special permission from environmental authorities is required for any intervention related to *Ae. albopictus*, as for example sampling and control measures. Legally, the Asian tiger mosquito requires an environmental management plan in order to reduce its impact and presence, as other introduced species such as the zebra mussel (*Dreissena polymorpha* Pallas, 1771). Unfortunately, to date there is not any national strategy for reducing the populations and dispersal of *Ae. albopictus* in Spain. But, we should bear on mind that according to the “One Health” approach, environmental management of invasive vector mosquito species is a key issue in public health concerns [74].

Regarding to the responsibility of controlling mosquito populations in public places in Spain, the municipalities are usually in the front line, but the legal basis of this competence is not clear or explicit, and only ambiguous references are made to “*obligation to maintain the public health*” in some Spanish, national and regional, laws. Some municipalities in provinces such as Barcelona, Castellón and Murcia, have adopted local ordinances which encourage residents to reduce larval habitats as well as to control *Ae. albopictus* populations in private properties. In Catalonia, there is also a proposal of by-law type for being adopted by municipalities. There are few isolated municipalities which have this type of ordinances, with different grade of legal restrictions, such as Sant Cugat del Vallès, Badalona, Montmeló and Barberà del Vallès (province of Barcelona), Torreblanca (prov. Castellón) and Mazarrón (prov. Murcia).

### Control measures

Just as surveillance, in Spain the control plans are not systematised or standardised. Many municipalities have outsourced the mosquitoes control, but the specific training and experience, on tiger mosquito control, of the awarded companies is very variable.

The main mosquito control programs have been developed in Catalonia. For example, in the Baix Llobregat comarca (county), the integrated vector management includes community-based information plans and application of larvicides and adulticides [75]. In Sant Cugat del Vallés, Abramides et al. [76] combined four control measures, including application of larvicides and adulticides, public cooperation for source removal and cleaning of landfills, finding a significant reduction of oviposition. From 2008 to 2010, a massive campaign for source-reduction was carried out in the same municipality, including door-to-door visits and interviews to residents [77]. This campaign allowed to inform the local population, to identify larval habitats, to measure resident’s perception of the problem and achieved the involvement of citizens in control. In this reference the more usual breeding sites in houses are described: domestic drains (27.8 %), man-made ponds or pools without chlorine (26.7 %), drums (25.6 %), solid waste (12.9 %), flowerpot saucers (9.8 %), small objects (toys, ashtrays, pet water bowl) (9.8 %), ornamental fountains (9.8 %), other receptacles (8.3 %). Also, in public spaces of Catalonia the storm drains are very important places for control not just because they are frequently positives, but also because contain many larvae by ml. Nevertheless, the first searches carried out in Murcia to locate larvae in the storm drains have been unsuccessful. Finally, non artificial breeding sites, as holes in trees, are very scarce, although it seems more abundant in the recent times, maybe due to the higher densities in Catalonia.

Also, some tests of biocides have been conducted in Spain. Bengoa et al. tested residual pyrethroids on plants to act as chemical barrier against adults [75] and ultra-low volume (ULV) method with pyrethroids [78]. Both tests resulted in effective outcomes. Eritja [79] successfully tested the combination of *Bacilus thuringensis israelensis* and *B. sphaericus*, VBC60035 (Vectomax™ FG), as larvicide in simulated catch basins, a common urban larval habitat. In 2013, a laboratory test with encapsulated adulticides (INESFLY®) was carried out in the University of Zaragoza and the three used formulations (5A IGR NG, 5A IGR, 5D IGR) (IGR = insect growth regulator) were 100 % effective [80]. This method is not used extensively against *Ae. albopictus* in Spain yet, although similar products of this company are used against other insects (http://www.inesfly.com/index.php/es/proyectos-inesfly-mundo/category/espana-2).

The oil of lemon eucalyptus was tested as larvicide for control of *Ae. albopictus,* showing not to be applicable due to its high toxicity for non-target aquatic species as mosquitofish and frog tadpoles [81].

## Conclusion

Ten years after the first record of *Ae. albopictus* this species is, unfortunately, well established and expanding along the Spanish Mediterranean Coast and we fear that there is a high risk that the Atlantic coast to be also colonized. As has been observed in several invaded countries, this species has high ecological adaptability and will settle down in more areas than it was expected, such as the early data of incomplete samplings of 2015 show. Increasing range of distribution, and positive localisations inside it, of *Ae. albopictus* in Spain (including the Balearic archipelago), as well as the high detection of imported cases of viruses such as dengue and chikingunya and other arboviruses, depict an alarming scenario where implementation of preventive and coordinated measures among all implicated actors appear as the most reasonable tool. Active and urgent reaction is therefore needed from central, regional and local governments to face the public health risk posed by the presence of the Asian tiger mosquito. In this sense, the future standardisation of sampling and control techniques among researchers, local administrations and policymakers, and extent them to entire territory with appropriate budget, favoured by the umbrella of a national strategy for managing *Ae. albopictus* would be, in our opinion, the main milestone for the next five years.

## Additional file

Additional file 1:
**Historical record of the first detections of** ***Aedes albopictus*** **in municipalities of Spain.** The bibliographical reference is included or it is considered as new record if this information was still unpublished. The reference number is related to the list of references of main document. (PDF 169 kb)
