# Supplementary material for: Review of ten-years presence of Aedes albopictus in Spain 2004–2014: known distribution and public health concerns
Source: Parasit Vectors. 2015 Dec 23;8:655. doi: 10.1186/s13071-015-1262-y (PMC4688962; doi:10.1186/s13071-015-1262-y)
Supplement: Additional file 1: — Historical record of the first detections of Aedes albopictus in municipalities of Spain. The bibliographical reference is included or it is considered as new record if this information was still unpublished. The reference number is related to the list of references of main document. (PDF 169 kb) [file 13071_2015_1262_MOESM1_ESM.pdf]

Appendix

Historical record of the first detections of *Aedes albopictus* in municipalities of Spain. The bibliographical reference is included or it is considered as new record if this information was still unpublished. The reference number is related to the list of references of main document.

Record year	Municipality	Province/Island	Region	Reference
2004	Cerdanyola del Vallès	Barcelona	Catalonia	[9]
2004	Sant Cugat del Vallès	Barcelona	Catalonia	[3]
2005	Barberà del Vallès	Barcelona	Catalonia	[11]
2005	Barcelona	Barcelona	Catalonia	[11]
2005	El Papiol	Barcelona	Catalonia	[11]
2005	Molins de Rei	Barcelona	Catalonia	[9]
2005	Ripollet	Barcelona	Catalonia	[9]
2005	Rubí	Barcelona	Catalonia	[9]
2005	Sant Quirze del Vallès	Barcelona	Catalonia	[9]
2005	Terrassa	Barcelona	Catalonia	[9]
2005	Altafulla	Tarragona	Catalonia	[11]
2005	Orihuela	Alicante	Valencian Community	[11]
2006	Badia del Vallés	Barcelona	Catalonia	[9]
2006	Caldes d'Estrac	Barcelona	Catalonia	[9]
2006	Castellbisbal	Barcelona	Catalonia	[9]
2006	Castelldefels	Barcelona	Catalonia	[11]
2006	Esplugues de Llobregat	Barcelona	Catalonia	[11]
2006	Gavà	Barcelona	Catalonia	[11]
2006	L'Hospitalet de Llobregat	Barcelona	Catalonia	[11]
2006	Montcada i Reixac	Barcelona	Catalonia	[9]
2006	Pallejà	Barcelona	Catalonia	[11]
2006	Polinyà	Barcelona	Catalonia	[9]
2006	Sabadell	Barcelona	Catalonia	[9]
2006	Sant Feliu de Llobregat	Barcelona	Catalonia	[11]
2006	Sant Just Desvern	Barcelona	Catalonia	[11]
2006	Santa Perpètua de Mogoda	Barcelona	Catalonia	[10]
2006	Sitges	Barcelona	Catalonia	[11]
2006	Vallirana	Barcelona	Catalonia	[11]
2006	Vilanova i la Geltrú	Barcelona	Catalonia	[11]
2007	Abrera	Barcelona	Catalonia	[10]
2007	Arenys de Mar	Barcelona	Catalonia	[10]
2007	Badalona	Barcelona	Catalonia	[10]
2007	Canet de Mar	Barcelona	Catalonia	[10]
2007	Castellar del Vallès	Barcelona	Catalonia	[10]
2007	Castellví de Rosanes	Barcelona	Catalonia	[10]
2007	Cervelló	Barcelona	Catalonia	[10]
2007	Cornellà de Llobregat	Barcelona	Catalonia	[10]
2007	Cubelles	Barcelona	Catalonia	[10]
2007	El Masnou	Barcelona	Catalonia	[10]
2007	El Prat de Llobregat	Barcelona	Catalonia	[10]
2007	Esparreguera	Barcelona	Catalonia	[10]
2007	Gelida	Barcelona	Catalonia	[10]

2007	La Palma de Cervelló	Barcelona	Catalonia	[10]
2007	Martorell	Barcelona	Catalonia	[10]
2007	Mollet del Vallès	Barcelona	Catalonia	[10]
2007	Montgat	Barcelona	Catalonia	[10]
2007	Palau-solità i Plegamans	Barcelona	Catalonia	[10]
2007	Sant Adrià de Besos	Barcelona	Catalonia	[10]
2007	Sant Andreu de la Barca	Barcelona	Catalonia	[10]
2007	Sant Boi de Llobregat	Barcelona	Catalonia	[10]
2007	Sant Fost de Campsentelles	Barcelona	Catalonia	[10]
2007	Sant Joan Despí	Barcelona	Catalonia	[10]
2007	Santa Coloma de Cervelló	Barcelona	Catalonia	[10]
2007	Santa Coloma de Gramenet	Barcelona	Catalonia	[10]
2007	Ullastrell	Barcelona	Catalonia	[10]
2007	Viladecans	Barcelona	Catalonia	[10]
2008	Blanes	Girona	Catalonia	[10]
2008	Lloret de Mar	Girona	Catalonia	[10]
2008	Maçanet de la Selva	Girona	Catalonia	[10]
2008	Tossa de Mar	Girona	Catalonia	[10]
2008	Vidreres	Girona	Catalonia	[10]
2008	Argentona	Barcelona	Catalonia	[10]
2008	Begues	Barcelona	Catalonia	[10]
2008	Calella	Barcelona	Catalonia	[10]
2008	Canyelles	Barcelona	Catalonia	[10]
2008	Cardedeu	Barcelona	Catalonia	[10]
2008	Granollers	Barcelona	Catalonia	[10]
2008	Lliçà d'Amunt	Barcelona	Catalonia	[10]
2008	Martorelles	Barcelona	Catalonia	[10]
2008	Premià de Dalt	Barcelona	Catalonia	[10]
2008	Premià de Mar	Barcelona	Catalonia	[10]
2008	Sant Andreu de Llavaneres	Barcelona	Catalonia	[10]
2008	Sant Cebrià de Vallalta	Barcelona	Catalonia	[10]
2008	Sant Esteve Sesrovires	Barcelona	Catalonia	[10]
2008	Sant Pere de Ribes	Barcelona	Catalonia	[10]
2008	Sant Vicenç de Castellet	Barcelona	Catalonia	[10]
2008	Sant Vicenç de Montalt	Barcelona	Catalonia	[10]
2008	Sant Vicenç dels Horts	Barcelona	Catalonia	[10]
2008	Teià	Barcelona	Catalonia	[10]
2008	Tiana	Barcelona	Catalonia	[10]
2008	Tordera	Barcelona	Catalonia	[10]
2008	Torrelles de Llobregat	Barcelona	Catalonia	[10]
2008	Vacarisses	Barcelona	Catalonia	[10]
2008	Viladecavalls	Barcelona	Catalonia	[10]
2008	Vilassar de Dalt	Barcelona	Catalonia	[10]
2008	Maials	Lleida	Catalonia	[10]
2008	Calafell	Tarragona	Catalonia	[10]
2008	El Vendrell	Tarragona	Catalonia	[10]
2008	Tarragona	Tarragona	Catalonia	[10]
2008	Torredembarra	Tarragona	Catalonia	[10]

2009	Caldes de Malavella	Girona	Catalonia	[13]
2009	Calonge	Girona	Catalonia	[13]
2009	Castell-Platja d'Aro	Girona	Catalonia	[13]
2009	L'Escala	Girona	Catalonia	[13]
2009	Llagostera	Girona	Catalonia	[13]
2009	Riudarenes	Girona	Catalonia	[13]
2009	Sils	Girona	Catalonia	[13]
2009	Alella	Barcelona	Catalonia	[13]
2009	Arenys de Munt	Barcelona	Catalonia	[13]
2009	Cabrera de Mar	Barcelona	Catalonia	[13]
2009	Caldes de Montbui	Barcelona	Catalonia	[13]
2009	Castellbell i el Vilar	Barcelona	Catalonia	[13]
2009	La Garriga	Barcelona	Catalonia	[13]
2009	Lliçà de Vall	Barcelona	Catalonia	[13]
2009	Manresa	Barcelona	Catalonia	[13]
2009	Masquefa	Barcelona	Catalonia	[13]
2009	Matadepera	Barcelona	Catalonia	[13]
2009	Mataró	Barcelona	Catalonia	[13]
2009	Montmeló	Barcelona	Catalonia	[13]
2009	Montornès del Vallès	Barcelona	Catalonia	[13]
2009	Olesa de Bonesvalls	Barcelona	Catalonia	[13]
2009	Parets del Vallès	Barcelona	Catalonia	[13]
2009	Piera	Barcelona	Catalonia	[13]
2009	Rellinars	Barcelona	Catalonia	[13]
2009	Sant Fruitós de Bages	Barcelona	Catalonia	[13]
2009	Sant Llorenç d'Hortons	Barcelona	Catalonia	[13]
2009	Sant Llorenç Savall	Barcelona	Catalonia	[13]
2009	Sant Sadurní d'Anoia	Barcelona	Catalonia	[13]
2009	Santa Eulàlia de Ronçana	Barcelona	Catalonia	[13]
2009	Santa Maria de Martorelles	Barcelona	Catalonia	[13]
2009	Subirats	Barcelona	Catalonia	[13]
2009	Vallromanes	Barcelona	Catalonia	[13]
2009	Vilafranca del Penedès	Barcelona	Catalonia	[13]
2009	Vilanova del Vallès	Barcelona	Catalonia	[13]
2009	Vilassar de Mar	Barcelona	Catalonia	[13]
2009	Creixell	Tarragona	Catalonia	[13]
2009	Roda de Barà	Tarragona	Catalonia	[13]
2009	Torrevieja	Alicante	Valencian Community	Delacour et al. 2009
2010	Girona	Girona	Catalonia	[13]
2010	Sant Andreu de Salou	Girona	Catalonia	[13]
2010	Avinyonet del Penedès	Barcelona	Catalonia	[13]
2010	Bigues i Riells	Barcelona	Catalonia	[13]
2010	Cabrera d'Anoia	Barcelona	Catalonia	[13]
2010	Cabrils	Barcelona	Catalonia	[13]
2010	Canovelles	Barcelona	Catalonia	[13]
2010	Cànoves i Samalús	Barcelona	Catalonia	[13]
2010	Castellet i la Gornal	Barcelona	Catalonia	New record

2010	Castellgalí	Barcelona	Catalonia	[13]
2010	Castellolí	Barcelona	Catalonia	[13]
2010	Collbató	Barcelona	Catalonia	New record
2010	Corbera de Llobregat	Barcelona	Catalonia	New record
2010	Dosrius	Barcelona	Catalonia	[13]
2010	El Bruc	Barcelona	Catalonia	[13]
2010	El Pont de Vilomara i Rocafort	Barcelona	Catalonia	[13]
2010	Els Hostalets de Pierola	Barcelona	Catalonia	[13]
2010	Gallifa	Barcelona	Catalonia	[13]
2010	L'Ametlla del Vallès	Barcelona	Catalonia	[13]
2010	La Llagosta	Barcelona	Catalonia	[13]
2010	Les Franqueses del Vallès	Barcelona	Catalonia	[13]
2010	Malgrat de Mar	Barcelona	Catalonia	[13]
2010	Marganell	Barcelona	Catalonia	[13]
2010	Monistrol de Montserrat	Barcelona	Catalonia	[13]
2010	Olesa de Montserrat	Barcelona	Catalonia	New record
2010	Olivella	Barcelona	Catalonia	[13]
2010	Òrrius	Barcelona	Catalonia	[13]
2010	Palafolls	Barcelona	Catalonia	[13]
2010	Pineda de Mar	Barcelona	Catalonia	[13]
2010	Sant Celoni	Barcelona	Catalonia	[13]
2010	Sant Climent de Llobregat	Barcelona	Catalonia	New record
2010	Sant Cugat Sesgarrigues	Barcelona	Catalonia	[13]
2010	Sant Feliu de Codines	Barcelona	Catalonia	[13]
2010	Sant Iscle de Vallalta	Barcelona	Catalonia	[13]
2010	Sant Pol de Mar	Barcelona	Catalonia	[13]
2010	Santa Susanna	Barcelona	Catalonia	[13]
2010	Sentmenat	Barcelona	Catalonia	[13]
2010	Torrelavit	Barcelona	Catalonia	[13]
2010	Vallbona d'Anoia	Barcelona	Catalonia	[13]
2010	Bellvei	Tarragona	Catalonia	[13]
2010	Cambrils	Tarragona	Catalonia	[13]
2010	Castellvell del Camp	Tarragona	Catalonia	[13]
2010	Cunit	Tarragona	Catalonia	[13]
2010	El Catllar	Tarragona	Catalonia	[13]
2010	Els Pallaresos	Tarragona	Catalonia	[13]
2010	La Pobla de Montornès	Tarragona	Catalonia	[13]
2010	La Riera de Gaià	Tarragona	Catalonia	[13]
2010	Reus	Tarragona	Catalonia	[13]
2010	Salou	Tarragona	Catalonia	[13]
2010	Vila-seca	Tarragona	Catalonia	[13]
2010	Benicàssim	Castellón	Valencian Community	[24]
2011	Aiguaviva	Girona	Catalonia	New record
2011	Banyoles	Girona	Catalonia	New record
2011	Begur	Girona	Catalonia	New record
2011	Cassà de la Selva	Girona	Catalonia	New record
2011	Castelló d'Empúries	Girona	Catalonia	New record
2011	Celrà	Girona	Catalonia	New record

2011	Figueres	Girona	Catalonia	New record
2011	Fornells de la Selva	Girona	Catalonia	New record
2011	Hostalric	Girona	Catalonia	New record
2011	La Jonquera	Girona	Catalonia	New record
2011	Palafrugell	Girona	Catalonia	New record
2011	Palamós	Girona	Catalonia	New record
2011	Pals	Girona	Catalonia	New record
2011	Riells i Viabrea	Girona	Catalonia	New record
2011	Riudellots de la Selva	Girona	Catalonia	New record
2011	Roses	Girona	Catalonia	New record
2011	Salt	Girona	Catalonia	New record
2011	Sant Feliu de Buixalleu	Girona	Catalonia	New record
2011	Sant Feliu de Guíxols	Girona	Catalonia	New record
2011	Sant Gregori	Girona	Catalonia	New record
2011	Sant Julià de Ramis	Girona	Catalonia	New record
2011	Santa Coloma de Farners	Girona	Catalonia	New record
2011	Santa Cristina d'Aro	Girona	Catalonia	New record
2011	Sarrià de Ter	Girona	Catalonia	New record
2011	Torroella de Montgrí	Girona	Catalonia	New record
2011	Vilablareix	Girona	Catalonia	New record
2011	Vilamalla	Girona	Catalonia	New record
2011	Vilobí d'Onyar	Girona	Catalonia	New record
2011	Aiguafreda	Barcelona	Catalonia	New record
2011	Artés	Barcelona	Catalonia	New record
2011	Capellades	Barcelona	Catalonia	New record
2011	Castellnou de Bages	Barcelona	Catalonia	New record
2011	Centelles	Barcelona	Catalonia	New record
2011	Fogars de la Selva	Barcelona	Catalonia	New record
2011	Fogars de Montclús	Barcelona	Catalonia	New record
2011	Fonollosa	Barcelona	Catalonia	New record
2011	Font-rubí	Barcelona	Catalonia	New record
2011	La Pobla de Claramunt	Barcelona	Catalonia	New record
2011	La Roca del Vallès	Barcelona	Catalonia	New record
2011	Mediona	Barcelona	Catalonia	New record
2011	Navarcles	Barcelona	Catalonia	New record
2011	Rajadell	Barcelona	Catalonia	New record
2011	Sant Antoni de Vilamajor	Barcelona	Catalonia	New record
2011	Sant Joan de Vilatorrada	Barcelona	Catalonia	New record
2011	Sant Quintí de Mediona	Barcelona	Catalonia	New record
2011	Santa Fe del Penedès	Barcelona	Catalonia	New record
2011	Santa Margarida i els Monjos	Barcelona	Catalonia	New record
2011	Santa Maria de Palautordera	Barcelona	Catalonia	New record
2011	Santpedor	Barcelona	Catalonia	New record
2011	Vallgorguina	Barcelona	Catalonia	New record
2011	Vilanova del Camí	Barcelona	Catalonia	New record
2011	Albinyana	Tarragona	Catalonia	New record
2011	Banyeres del Penedès	Tarragona	Catalonia	New record
2011	Bonastre	Tarragona	Catalonia	New record

2011	Constantí	Tarragona	Catalonia	New record
2011	L'Arboç	Tarragona	Catalonia	New record
2011	La Bisbal de Penedés	Tarragona	Catalonia	New record
2011	La Nou de Gaià	Tarragona	Catalonia	New record
2011	La Secuita	Tarragona	Catalonia	New record
2011	La Selva del Camp	Tarragona	Catalonia	New record
2011	Les Borges del Camp	Tarragona	Catalonia	New record
2011	Llorenç del Penedès	Tarragona	Catalonia	New record
2011	Maspujols	Tarragona	Catalonia	New record
2011	Mont-roig del Camp	Tarragona	Catalonia	New record
2011	Montbrió del Camp	Tarragona	Catalonia	New record
2011	Perafort	Tarragona	Catalonia	New record
2011	Riudoms	Tarragona	Catalonia	New record
2011	Santa Oliva	Tarragona	Catalonia	New record
2011	Valls	Tarragona	Catalonia	New record
2011	Vinyols i els Arcs	Tarragona	Catalonia	New record
2011	Orpesa	Castellón	Valencian Community	[33]
2011	Guardamar del Segura	Alicante	Valencian Community	[33]
2011	Pilar de la Horadada	Alicante	Valencian Community	[33]
2011	Murcia	Murcia	Region of Murcia	[31]
2012	Anglès	Girona	Catalonia	New record
2012	Bescanó	Girona	Catalonia	New record
2012	Breda	Girona	Catalonia	New record
2012	Camós	Girona	Catalonia	New record
2012	Darnius	Girona	Catalonia	New record
2012	Olot	Girona	Catalonia	New record
2012	Porqueres	Girona	Catalonia	New record
2012	Vilafant	Girona	Catalonia	New record
2012	Avinyó	Barcelona	Catalonia	New record
2012	Callús	Barcelona	Catalonia	New record
2012	Carme	Barcelona	Catalonia	New record
2012	Figaró - Montmany	Barcelona	Catalonia	New record
2012	Gualba	Barcelona	Catalonia	New record
2012	Igualada	Barcelona	Catalonia	New record
2012	La Llacuna	Barcelona	Catalonia	New record
2012	Les Cabanyes	Barcelona	Catalonia	New record
2012	Llinars del Vallès	Barcelona	Catalonia	New record
2012	Olèrdola	Barcelona	Catalonia	New record
2012	Pacs del Penedès	Barcelona	Catalonia	New record
2012	Pontons	Barcelona	Catalonia	New record
2012	Puigdàlber	Barcelona	Catalonia	New record
2012	Sallent	Barcelona	Catalonia	New record
2012	Tagamanent	Barcelona	Catalonia	New record
2012	Torrelles de Foix	Barcelona	Catalonia	New record
2012	Vic	Barcelona	Catalonia	New record
2012	Vilobí del Penedès	Barcelona	Catalonia	New record
2012	Alcover	Tarragona	Catalonia	New record
2012	Botarell	Tarragona	Catalonia	New record

2012	Bràfim	Tarragona	Catalonia	New record
2012	El Montmell	Tarragona	Catalonia	New record
2012	El Morell	Tarragona	Catalonia	New record
2012	Els Garidells	Tarragona	Catalonia	New record
2012	L'Aleixar	Tarragona	Catalonia	New record
2012	La Canonja	Tarragona	Catalonia	New record
2012	La Pobla de Mafumet	Tarragona	Catalonia	New record
2012	Masllorenç	Tarragona	Catalonia	New record
2012	Montferri	Tarragona	Catalonia	New record
2012	Nulles	Tarragona	Catalonia	New record
2012	Renau	Tarragona	Catalonia	New record
2012	Rodonyà	Tarragona	Catalonia	New record
2012	Salomó	Tarragona	Catalonia	New record
2012	Sant Jaume dels Domenys	Tarragona	Catalonia	New record
2012	Vallmoll	Tarragona	Catalonia	New record
2012	Vespella de Gaià	Tarragona	Catalonia	New record
2012	Vilallonga del Camp	Tarragona	Catalonia	New record
2012	Vilanova d'Escornalbou	Tarragona	Catalonia	New record
2012	Borriol	Castellón	Valencian Community	[33]
2012	Castelló de la Plana	Castellón	Valencian Community	[33]
2012	La Pobla de Tornessa	Castellón	Valencian Community	[33]
2012	Torreblanca	Castellón	Valencian Community	[33]
2012	Vall d´Alba	Castellón	Valencian Community	[33]
2012	Benijófar	Alicante	Valencian Community	[33]
2012	Elx	Alicante	Valencian Community	[33]
2012	Rojales	Alicante	Valencian Community	[33]
2012	Santa Pola	Alicante	Valencian Community	[33]
2012	Bunyola	Balearic/Majorca	Balearic Islands	[32]
2012	Calvià	Balearic/Majorca	Balearic Islands	[32]
2012	Esporles	Balearic/Majorca	Balearic Islands	[32]
2012	Marratxí	Balearic/Majorca	Balearic Islands	[32]
2012	Palma de Mallorca	Balearic/Majorca	Balearic Islands	[32]
2012	Mazarrón	Murcia	Region of Murcia	[14]
2012	San Pedro del Pinatar	Murcia	Region of Murcia	[33]
2013	Massanes	Girona	Catalonia	New record
2013	Balsareny	Barcelona	Catalonia	New record
2013	Campins	Barcelona	Catalonia	New record
2013	Castellfollit del Boix	Barcelona	Catalonia	New record
2013	El Pla del Penedès	Barcelona	Catalonia	New record
2013	Navàs	Barcelona	Catalonia	New record
2013	Sant Pere de Vilamajor	Barcelona	Catalonia	New record
2013	Sant Quirze Safaja	Barcelona	Catalonia	New record
2013	Santa Margarida de Montbui	Barcelona	Catalonia	New record
2013	Súria	Barcelona	Catalonia	New record
2013	Talamanca	Barcelona	Catalonia	New record
2013	Alió	Tarragona	Catalonia	New record
2013	Almoster	Tarragona	Catalonia	New record
2013	El Milà	Tarragona	Catalonia	New record

2013	El Rourell	Tarragona	Catalonia	New record
2013	La Masó	Tarragona	Catalonia	New record
2013	Montblanc	Tarragona	Catalonia	New record
2013	Pla de Santa Maria	Tarragona	Catalonia	New record
2013	Pratdip	Tarragona	Catalonia	New record
2013	Puigpelat	Tarragona	Catalonia	New record
2013	Vandellòs i l'Hospitalet de l'Infant	Tarragona	Catalonia	New record
2013	Vila-rodona	Tarragona	Catalonia	New record
2013	Alcalà de Xivert	Castellón	Valencian Community	[29]
2013	Almassora	Castellón	Valencian Community	[29]
2013	Benicarló	Castellón	Valencian Community	[29]
2013	Benlloch	Castellón	Valencian Community	[29]
2013	Borriana	Castellón	Valencian Community	[29]
2013	Cabanes	Castellón	Valencian Community	[29]
2013	L'Alcora	Castellón	Valencian Community	[29]
2013	La Serra d´en Galceran	Castellón	Valencian Community	[29]
2013	Les Coves de Vinromà	Castellón	Valencian Community	[29]
2013	Peñiscola	Castellón	Valencian Community	[29]
2013	Sant Juan de Moró	Castellón	Valencian Community	[29]
2013	Vila-real	Castellón	Valencian Community	[29]
2013	Vilafamés	Castellón	Valencian Community	[29]
2013	Vilanova d´Alcolea	Castellón	Valencian Community	[29]
2013	Vinarós	Castellón	Valencian Community	[29]
2013	Cullera	Valencia	Valencian Community	[29]
2013	Gandia	Valencia	Valencian Community	[29]
2013	Real de Gandia	Valencia	Valencian Community	[15]
2013	Sueca	Valencia	Valencian Community	[29]
2013	Algorfa	Alicante	Valencian Community	[29]
2013	Alicante	Alicante	Valencian Community	[29]
2013	Benejúzar	Alicante	Valencian Community	[29]
2013	Daia Nova	Alicante	Valencian Community	[29]
2013	Daia Vella	Alicante	Valencian Community	[29]
2013	Dolores	Alicante	Valencian Community	[29]
2013	El Campello	Alicante	Valencian Community	[17]
2013	Formentera del Segura	Alicante	Valencian Community	[29]
2013	Jacarilla	Alicante	Valencian Community	[29]
2013	Los Montesinos	Alicante	Valencian Community	[29]
2013	San Fulgencio	Alicante	Valencian Community	[29]
2013	Banyalbufar	Balearic/Majorca	Balearic Islands	New record
2013	Santa Maria del Camí	Balearic/Majorca	Balearic Islands	New record
2013	Águilas	Murcia	Region of Murcia	[29]
2013	Cartagena	Murcia	Region of Murcia	[29]
2013	San Javier	Murcia	Region of Murcia	[29]
2014	Amer	Girona	Catalonia	New record
2014	Besalú	Girona	Catalonia	New record
2014	Bordils	Girona	Catalonia	New record
2014	Brunyola	Girona	Catalonia	New record
2014	Campllong	Girona	Catalonia	New record

2014	Canet d'Adri	Girona	Catalonia	New record
2014	Cruïlles, Monells i St Sadurní de l'Heura	Girona	Catalonia	New record
2014	Forallac	Girona	Catalonia	New record
2014	Juià	Girona	Catalonia	New record
2014	La Cellera de Ter	Girona	Catalonia	New record
2014	Llambilles	Girona	Catalonia	New record
2014	Mieres	Girona	Catalonia	New record
2014	Mont-ras	Girona	Catalonia	New record
2014	Quart	Girona	Catalonia	New record
2014	Sant Joan de Mollet	Girona	Catalonia	New record
2014	Sant Julià de Llor i Bonmatí	Girona	Catalonia	New record
2014	Sant Martí Vell	Girona	Catalonia	New record
2014	Vall-llobrega	Girona	Catalonia	New record
2014	Aguilar de Segarra	Barcelona	Catalonia	New record
2014	Berga	Barcelona	Catalonia	New record
2014	Calders	Barcelona	Catalonia	New record
2014	Castellterçol	Barcelona	Catalonia	New record
2014	Castellví de la Marca	Barcelona	Catalonia	New record
2014	La Torre de Claramunt	Barcelona	Catalonia	New record
2014	Monistrol de Calders	Barcelona	Catalonia	New record
2014	Òdena	Barcelona	Catalonia	New record
2014	Orpí	Barcelona	Catalonia	New record
2014	Sant Esteve de Palautordera	Barcelona	Catalonia	New record
2014	Sant Martí de Tous	Barcelona	Catalonia	New record
2014	Sant Martí Sarroca	Barcelona	Catalonia	New record
2014	Sant Pere Riudebitlles	Barcelona	Catalonia	New record
2014	Santa Maria d'Oló	Barcelona	Catalonia	New record
2014	Vilalba Sasserra	Barcelona	Catalonia	New record
2014	Aiguamúrcia	Tarragona	Catalonia	New record
2014	Alcanar	Tarragona	Catalonia	New record
2014	Alforja	Tarragona	Catalonia	New record
2014	Amposta	Tarragona	Catalonia	New record
2014	Cabra del Camp	Tarragona	Catalonia	New record
2014	Camarles	Tarragona	Catalonia	New record
2014	Deltebre	Tarragona	Catalonia	New record
2014	Duesaigües	Tarragona	Catalonia	New record
2014	Figuerola del Camp	Tarragona	Catalonia	New record
2014	L'Aldea	Tarragona	Catalonia	New record
2014	L'Ametlla de Mar	Tarragona	Catalonia	New record
2014	L'Ampolla	Tarragona	Catalonia	New record
2014	L'Espluga de Fancolí	Tarragona	Catalonia	New record
2014	La Riba	Tarragona	Catalonia	New record
2014	Riudecanyes	Tarragona	Catalonia	New record
2014	Riudecols	Tarragona	Catalonia	New record
2014	Roquetes	Tarragona	Catalonia	New record
2014	Sant Carles de la Ràpita	Tarragona	Catalonia	New record
2014	Sant Jaume d'Enveja	Tarragona	Catalonia	New record

2014	Tivissa	Tarragona	Catalonia	New record
2014	Tortosa	Tarragona	Catalonia	New record
2014	Vilabella	Tarragona	Catalonia	New record
2014	Vilaplana	Tarragona	Catalonia	New record
2014	Vilaverd	Tarragona	Catalonia	New record
2014	Vimbodí i Poblet	Tarragona	Catalonia	New record
2014	Nules	Castellón	Valencian Community	New record
2014	Alzira	Valencia	Valencian Community	New record
2014	Canet d´en Berenguer	Valencia	Valencian Community	New record
2014	Godella	Valencia	Valencian Community	New record
2014	La Pobla de Farnals	Valencia	Valencian Community	New record
2014	Oliva	Valencia	Valencian Community	New record
2014	Paiporta	Valencia	Valencian Community	New record
2014	Picanya	Valencia	Valencian Community	[16]
2014	Sagunt	Valencia	Valencian Community	New record
2014	Tavernes de la Valldigna	Valencia	Valencian Community	New record
2014	València	Valencia	Valencian Community	New record
2014	Altea	Alicante	Valencian Community	New record
2014	Benidorm	Alicante	Valencian Community	New record
2014	Calp	Alicante	Valencian Community	New record
2014	Dènia	Alicante	Valencian Community	[18]
2014	Ondara	Alicante	Valencian Community	New record
2014	Sant Joan d´Alacant	Alicante	Valencian Community	New record
2014	Teulada	Alicante	Valencian Community	New record
2014	Villajoyosa	Alicante	Valencian Community	New record
2014	Xábia	Alicante	Valencian Community	New record
2014	Algaida	Balearic/Majorca	Balearic Islands	New record
2014	Andratx	Balearic/Majorca	Balearic Islands	New record
2014	Llucmajor	Balearic/Majorca	Balearic Islands	New record
2014	Puigpunyent	Balearic/Majorca	Balearic Islands	New record
2014	Santa Maria del Camí	Balearic/Majorca	Balearic Islands	New record
2014	Sant Antoni de Portmani	Balearic/Ibiza	Balearic Islands	[34]
2014	Alcantarilla	Murcia	Region of Murcia	New record
2014	Alhama De Murcia	Murcia	Region of Murcia	New record
2014	Beniel	Murcia	Region of Murcia	New record
2014	Fuente Álamo	Murcia	Region of Murcia	New record
2014	Las Torres de Cotillas	Murcia	Region of Murcia	New record
2014	Los Alcázares	Murcia	Region of Murcia	New record
2014	Molina de Segura	Murcia	Region of Murcia	New record
2014	Torre Pacheco	Murcia	Region of Murcia	New record
2014	Garrucha	Almería	Andalusia	New record
2014	Pulpí	Almería	Andalusia	New record
2014	Gualchos	Granada	Andalusia	New record
2014	Polopos	Granada	Andalusia	New record
2014	Alhaurín de la Torre	Málaga	Andalusia	[27]
2014	Irún	Guipúzcoa	Basque Country	[25]
